# Analysis of *Myxococcus xanthus* Vegetative Biofilms With Microtiter Plates

**DOI:** 10.3389/fmicb.2022.894562

**Published:** 2022-04-29

**Authors:** Keane J. Dye, Zhaomin Yang

**Affiliations:** Department of Biological Sciences, Virginia Tech, Blacksburg, VA, United States

**Keywords:** *Myxococcus xanthus*, vegetative biofilms, exopolysaccharide (EPS), type IV pilus (T4P), microplate assay

## Abstract

The bacterium *Myxococcus xanthus* forms both developmental and vegetative types of biofilms. While the former has been studied on both agar plates and submerged surfaces, the latter has been investigated predominantly on agar surfaces as swarming colonies. Here we describe the development of a microplate-based assay for the submerged biofilms of *M. xanthus* under vegetative conditions. We examined the impacts of inoculation, aeration, and temperature to optimize the conditions for the assay. Aeration was observed to be critical for the effective development of submerged biofilms by *M. xanthus*, an obligate aerobic bacterium. In addition, temperature plays an important role in the development of *M. xanthus* submerged biofilms. It is well established that the formation of submerged biofilms by many bacteria requires both exopolysaccharide (EPS) and the type IV pilus (T4P). EPS constitutes part of the biofilm matrix that maintains and organizes bacterial biofilms while the T4P facilitates surface attachment as adhesins. For validation, we used our biofilm assay to examine a multitude of *M. xanthus* strains with various EPS and T4P phenotypes. The results indicate that the levels of EPS, but not of piliation, positively correlate with submerged biofilm formation in *M. xanthus*.

## Introduction

Biofilms are surface-associated multicellular microbial communities that are prevalent in the natural environment (Flemming et al., 2016; Flemming and Wuertz, 2019). There are three types of bacterial biofilms that have been investigated (Mikkelsen et al., 2007; Armitano et al., 2014; Kovacs and Dragos, 2019; Sanchez-Vizuete et al., 2022). These include the colony-type biofilms (CBFs) that form on agar surfaces, the pellicle-type biofilms (PBFs) that form at liquid-air interfaces and the submerged-type biofilms (SBFs) that form on solid surfaces under aqueous submersion. Most widely studied among them is the SBF, thanks in no small part to the availability of a microplate-based assay for such biofilms (Christensen et al., 1985). This streamlined assay has facilitated the mechanistic study of many facets of SBF development, including cell attachment and regulation (Genevaux et al., 1996; O’Toole and Kolter, 1998; Lei et al., 2018). In this assay, biofilms are first allowed to develop on the submerged surface of a microwell. Afterward, the total biomass of the SBFs are stained with crystal violet (CV) and quantified by CV absorbance (*A*_*cv*_) measured by microplate readers. Such CV-retention assays can be performed in a high throughput format, greatly facilitating the analysis and studies of SBF formation of many bacterial species. These studies revealed that there are four distinct stages in the formation of bacterial SBFs (Costerton et al., 1995). Initially, planktonic cells in an aqueous environment, whether passively adrift or actively motile, recognize and begin to attach to a submerged surface using adhesins including the bacterial Type IV pilus (T4P) (Landini et al., 2010; Li et al., 2012; Ellison et al., 2017). Attached cells may propagate to form microcolonies on the surface with the simultaneous production of exopolysaccharide (EPS) and other biofilm matrix materials (Maunders and Welch, 2017). At this stage, bacterial species capable of active motility may cease their locomotion and transition to a state of sessility (Landini et al., 2010). As cell density and EPS production increase, microcolonies grow and eventually develop into mature SBFs wherein cells are afforded increased protection against desiccation, predation, and antimicrobial compounds (Flemming et al., 2016; Flemming and Wuertz, 2019). Lastly, cells within biofilms may disperse or escape and re-enter the planktonic state for dissemination (Guilhen et al., 2017). Given the advantages of life within a biofilm against the elements, it is no surprise that 80% or more of all bacterial cells on Earth are estimated to reside within biofilms (Flemming and Wuertz, 2019).

*Myxococcus xanthus* is a surface-motile and developmental bacterium that has evolved to live and move on damp or wet surfaces of soil particles (Zusman et al., 2007; Konovalova et al., 2010; Zhang et al., 2012). Its gliding motility allows the bacterium to move or translocate on moist solid surfaces (Mauriello et al., 2010). Under nutrient rich conditions, *M. xanthus* cells on agar surfaces form vegetative biofilms that grow and spread as moving carpets with a killer instinct (Mauriello et al., 2010; Munoz-Dorado et al., 2016). This is because *M. xanthus* is a predatory bacterium that consumes other bacteria by swarming over them as social groups (Berleman and Kirby, 2009; Thiery and Kaimer, 2020; Sydney et al., 2021). When nutrients or prey become limiting, *M. xanthus* initiates a well-orchestrated developmental program, leading to the formation of multicellular fruiting bodies wherein cells differentiate into non-motile and metabolically dormant myxospores (Bretl and Kirby, 2016; Munoz-Dorado et al., 2016; Popp and Mascher, 2019). Integral to this program are inter- and intra-cellular signal transduction systems that allow the coordinated movement of *M. xanthus* to form mound-like aggregates, each containing hundreds of thousands of cells (Shimkets, 1986; Velicer and Vos, 2009; Bretl and Kirby, 2016; Mercier and Mignot, 2016; Kroos, 2017). These aggregates eventually maturate into fruiting bodies with differentiated myxospores. The sporulation process is accomplished through regulated gene expression accompanied by cellular morphogenesis such that rod-shaped vegetative cells become spherical myxospores within fruiting bodies (O’Connor and Zusman, 1991a,b; Cao et al., 2015; Kroos, 2017).

The process of *M. xanthus* fruiting body formation, considered an elaborate form of bacterial biofilm development (O’Toole et al., 2000; van Gestel et al., 2015), has been observed and analyzed extensively for over a century (Thaxter, 1892, 1897; Bretl and Kirby, 2016; Munoz-Dorado et al., 2016; Kroos, 2017). These developmental biofilms have been studied both on agar plates as well as on submerged surfaces (Kuner and Kaiser, 1982; Shimkets, 1986; Bretl and Kirby, 2016; Keane and Berleman, 2016). In contrast, the vegetative biofilms of *M. xanthus* have been investigated almost exclusively as swarming colonies or CBFs with a heavy focus on motility and taxis (Zusman et al., 2007; Islam and Mignot, 2015; Munoz-Dorado et al., 2016; Wadhwa and Berg, 2021). These studies have uncovered that *M. xanthus* possesses two genetically distinct forms of locomotion known as the social (S) and the adventurous (A) gliding motility (Hodgkin and Kaiser, 1979). It is known that S motility is powered by the recurrent cycles of T4P extension and retraction like twitching motility in other bacteria (Kaiser, 1979; Lu et al., 2005; Zusman et al., 2007; Yang et al., 2014; Wadhwa and Berg, 2021). The mechanism for this form of motility is sometimes referred to as the “grappling hook” mechanism (Merz and Forest, 2002). In the current model, the distal end of an extended T4P attaches to a solid anchor, the ensuing retraction of the pilus then pulls the cell forward (Zusman et al., 2007; Mauriello et al., 2010; Yang et al., 2014; Wadhwa and Berg, 2021). In *M. xanthus*, the EPS deposited on substratum or associated with other cells is the preferred anchor for T4P attachment, explaining the social nature of T4P-mediated motility in *M. xanthus* (Li et al., 2003; Nudleman and Kaiser, 2004; Yang et al., 2014; Zhou and Nan, 2017). That is, EPS produced by other cells enhance or facilitate the movement of their kin cells in physical proximity. In contrast, the A motility system enables individual and isolated cells to translocate without the requirement of a neighboring cell (Hodgkin and Kaiser, 1979; Islam and Mignot, 2015; Nan and Zusman, 2016). The proposed mechanism for A motility involves a supramolecular motility machinery extending from the cytoplasm to the exterior (Nan et al., 2014; Jakobczak et al., 2015; Faure et al., 2016). On the cytoplasmic side, this machinery is connected to and travels on a prokaryotic cytoskeleton (Fu et al., 2018). On the outside, it can be anchored to a gliding surface at stationary focal adhesion sites (FASs) for force generation to move cells forward (Islam and Mignot, 2015; Faure et al., 2016; Nan and Zusman, 2016; Wadhwa and Berg, 2021). There is no doubt that the studies of swarming CBFs have led to significant insights into the motility mechanisms of *M. xanthus*. On the other hand, the nearly exclusive focus on motility in these studies has left the SBFs of vegetative *M. xanthus* to be an understudied area of research.

Formation of bacterial SBFs can be conveniently analyzed by a microtiter plate-based assay that has been applied to numerous bacterial species (Christensen et al., 1985; O’Toole and Kolter, 1998; Merritt et al., 2005; Kwasny and Opperman, 2010; Coffey and Anderson, 2014). In such assays, cell cultures are first inoculated into wells of a microtiter plate. SBFs are then allowed to develop on the submerged surfaces of the microwells under static conditions. Biofilms are subsequently quantified by CV staining after the removal of unattached cells that are not part of the SBF. Previously, a microplate-based protocol was used to study SBF formation of yellow and tan variants of *M. xanthus* (Dahl et al., 2011). In this protocol, henceforth referred to as the Dahl protocol, *M. xanthus* cells suspended in growth media at a high cell density were used to inoculate a microtiter plate which was then incubated overnight to seed the wells under static conditions. After this overnight incubation, the microwells were washed and replenished with fresh media to allow SBF development for 24 h before the CV-based quantification. Overnight seeding is generally not included in biofilm assays for other bacteria (Christensen et al., 1985; O’Toole and Kolter, 1998; Merritt et al., 2005; Kwasny and Opperman, 2010; Coffey and Anderson, 2014). Among other considerations, we wondered if the more conventional assay without an extra seeding step could be applied to analyze *M. xanthus* biofilm formation under vegetative conditions.

Here we report the development and adaptation of a 96-well microplate-based assay for the studies of vegetative SBFs of *M. xanthus*. We show that *M. xanthus* biofilms can be analyzed without the overnight seeding in the Dahl protocol (Dahl et al., 2011). During the optimization of the protocol, we uncovered that aeration is critical for the formation of SBFs by *M. xanthus*, which is an obligate aerobe. That is, SBF formation by *M. xanthus* is greatly enhanced by rotary shaking over static conditions. We applied our assay to selected strains with altered T4P and EPS phenotypes as a means of validation. Our results demonstrate that the formation of vegetative SBFs tightly correlates with the level of EPS but not of T4P in *M. xanthus*. The availability of this assay may facilitate the mechanistic studies of SBF formation in *M. xanthus*, a surfaced-adapted and obligate aerobe that is uniquely motile on and adherent to solid surfaces in its natural environment.

## Materials and Methods

### Strains, Growth Conditions, and Chemicals

The *M. xanthus* strains used in this study are listed in Table 1. Unless otherwise specified, all *M. xanthus* strains were grown and maintained on Casitone-yeast extract (CYE) agar plates or in CYE liquid media (Campos et al., 1978) at 32°C on a rotary shaker at 300 rotations per minute (RPM). A stock solution of 1% (wt/vol) CV (ACROS Chemicals) was prepared in 20% (vol/vol) ethanol (Decon Laboratories). Glacial acetic acid (Fisher) was used to make a 30% (vol/vol) acetic acid solution. The MOPS buffer contains 10 mM morpholinepropanesulfonic acid (pH 7.6) and 2 mM MgSO_4_.

**TABLE 1:** *Myxococcus xanthus* strains used in this study.

Strains	Genotype	Source/references
DK1622	WT	Kaiser, 1979
DK10416	Δ*pilB*	Wu et al., 1997
DK10409	Δ*pilT*	Wu et al., 1997
YZ603	Δ*difE*	Black and Yang, 2004
YZ604	Δ*difG*	Black and Yang, 2004
YZ613	Δ*difD*	Black and Yang, 2004
YZ641	Δ*difD* Δ*difG*	Black et al., 2006
YZ646	Δ*difD* Δ*difG* Δ*pilA*	Black et al., 2006
YZ690	Δ*pilA*	Black et al., 2017

### Biofilm Assays

The clear tissue culture (TC)-treated flat-bottom 96-well microplates (Falcon) were used for the development of *M. xanthus* SBFs per the Dahl protocol (Dahl et al., 2011) or according to the procedures as described later in the manuscript. For the Dahl protocol, *M. xanthus* cells in the logarithmic growth phase were harvested and resuspended in CYE media to an optical density at 600 nm (OD_600_) of 0.8. 100 μl aliquots of the cell suspension in quadruplicate were added to the wells of a microplate. After incubation at 28°C for 12 h under static conditions for overnight seeding, the media was removed and the wells were washed with the MOPS buffer. For biofilm development, 100 μl of fresh CYE was added to each well and the microplate was incubated under static conditions for 24 h at 32°C. To develop our protocol, *M. xanthus* cells in logarithmic growth were harvested and resuspended in CYE media to various OD_600_ as indicated in the text. Aliquots of 75, 100, or 125 μl of the cell suspensions were dispensed into the microwells in quadruplicate. Biofilms were allowed to develop at 32 or 27°C for 24 h under static conditions or on a rotary shaker at 230 RPM.

The SBFs developed above were quantified by the widely adopted CV-based method (Christensen et al., 1985; Kwasny and Opperman, 2010; Xi and Wu, 2010; Dahl et al., 2011; Redder and Linder, 2012; Naher et al., 2014; Bordeleau et al., 2018). Briefly, after the production of SBFs in microwells, the media and unattached cells were gently removed by a multichannel pipette. The wells were washed with equal volumes of MOPS buffer to the culture volume. Staining was conducted with 150 μl of 1% CV solution for 20 min before washing thrice with 175 μl of H_2_O. After air drying, 200 μl of 30% acetic acid was added to each well and incubated for 20 min. 125 μl of the acetic acid solution was then transferred to the microwells of a clear polystyrene 96-well microplate (ExtraGene). The CV absorbance (*A*_*cv*_) was measured at 600 nm using an Infinite F200 PRO plate reader and the *A*_*cv*_ values were used as the quantifier of SBF amounts per well. For some experiments, the biofilm amounts (*A*_*cv*_ values) were normalized to either the total area of the submerged surfaces or the final OD_600_ of the samples. The submerged surface area for a given sample was calculated from the culture volume in a microwell based on the specifications of the microtiter plate by the manufacturer. The submerged surface areas for the 75 μl, the 100 μl, and the 125 μl samples were determined to be 0.79, 0.95, and 1.10 cm^2^, respectively. To normalize SBF amount to cell density in a microwell, the OD_600_ of the cell culture after SBF development was measured 16 times by the Multiple Reads function of the plate reader. The average of these measurements was given as the final OD_600_. In some instances, Grubbs’ test identified one of the quadruple samples as an outlier which was expunged from the dataset. Statistical differences were determined using the Student’s *t*-test.

The linear range of the F200 PRO under our experimental conditions was determined by the measuring *A*_*cv*_ of serial dilutions of a CV solution. In total, 15 concentrations from 0 to 100 ppm were analyzed in quadruplicates in a 96-well microplate (Supplementary Figure 1). This analysis showed the linearity of *A*_*cv*_ vs. CV concentration extends up to 60 ppm of CV and *A*_*cv*_ values above 3.0. The coefficient of determination or *R*^2^ values are 0.9977 and 0.9999 for the CV concentration range of 0–60 ppm and of 0–30 ppm, respectively. When CV concentrations were higher than 60 ppm and the *A*_*cv*_ values went above 3.5, the linear relationship no longer holds (data not shown).

## Results and Discussion

### Direct Inoculation for *Myxococcus xanthus* Submerged Biofilm Development

**FIGURE 1:**
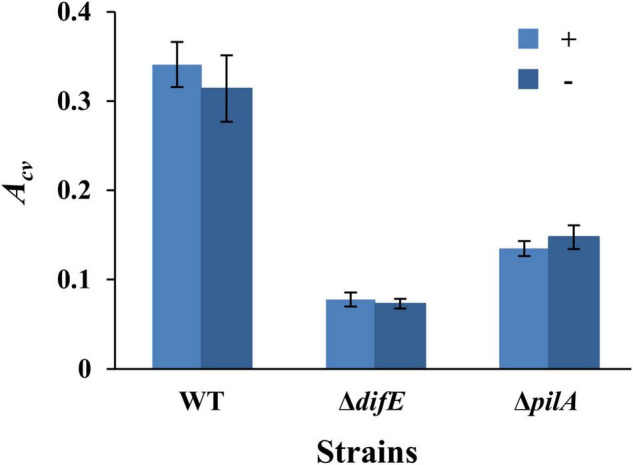
*Myxococcus xanthus* SBFs with or without overnight seeding. Shown are the SBF amounts represented by average *A*_*cv*_ values with standard deviations from three independent experiments, each conducted in quadruplicates. SBFs were formed with (+) or without (-) overnight seeding. The strains were DK1622 (WT), YZ603 (Δ*difE*), and YZ690 (Δ*pilA*).

We first examined if the conventional microplate-based biofilm assay (O’Toole and Kolter, 1998) without the overnight seeding step (Dahl et al., 2011) could be applied to *M. xanthus* SBFs under vegetative growth. For this, we compared the results from two sets of experiments. The first set was conducted according to the Dahl protocol such that a microwell was inoculated with 100 μl of *M. xanthus* cells at OD_600_ of 0.8 and incubated overnight. The microwell was then washed and replenished with fresh media to allow SBF development for 24 h at 32°C. For the second set, each well of the microtiter plate was inoculated with 100 μl of a cell suspension at OD_600_ of 0.1 and SBFs were allowed to develop directly for 24 h at 32°C. Three *M. xanthus* strains were used for the initial experiments: the wild type (WT) DK1622, the EPS^–^ strain YZ603 (Δ*difE*) and the T4P^–^ strain YZ690 (Δ*pilA*). It should be noted that the T4P^–^ strain is also deficient in EPS production because T4P is required for wild-type levels of EPS production in *M. xanthus* (Black et al., 2006). As shown in Figure 1, these two sets of experiments yielded similar trends of SBF formation for these strains. In both protocols, the WT produced significantly more biofilms than the EPS^–^ and T4P^–^ strains as reflected by CV absorbance (*A*_*cv*_). These trends were expected because both EPS and T4P have been demonstrated to be critical for biofilm formation in multiple bacteria (Bahar et al., 2009; Colvin et al., 2011; Maunders and Welch, 2017; Fiebig, 2019). Moreover, the amounts of SBFs as quantified by CV retention were comparable for the two protocols for all three strains. The *A*_*cv*_ values for the WT were 0.34 ± 0.03 and 0.31 ± 0.04 in these two protocols, respectively. Those for the Δ*difE* strains were 0.08 ± 0.01 and 0.07 ± 0.01, and the Δ*pilA* strain, 0.13 ± 0.01 and 0.15 ± 0.01, respectively. These observations indicate that a protocol without the seeding step performed comparably with the Dahl protocol. Overnight seeding was therefore eliminated from experimental procedures for the remainder of this study.

### Static Conditions May Limit Oxygen Availability to Impact *Myxococcus xanthus* Submerged-Type Biofilm Formation

**FIGURE 2:**
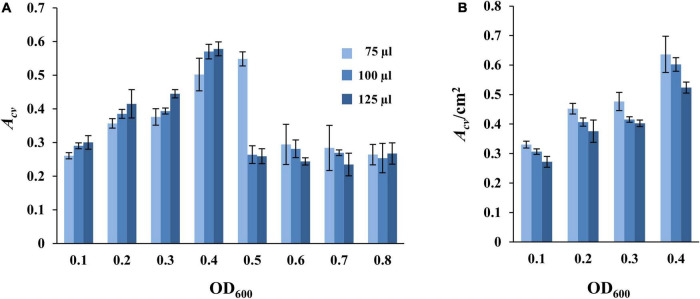
Impact of culture volume and cell density on static SBF formation. Different volumes of DK1622 (WT) cells at starting OD_600_ as indicated were inoculated into microwells for biofilm development at 32°C under static conditions. The amount of SBF per microwell was represented either by *A*_*cv*_ **(A)** or by *A*_*cv*_/cm^2^ **(B)** which is normalized to the submerged surface area as described in section “Materials and Methods.” Samples with starting OD_600_ of 0.5 or higher are not included in panel **(B)** due to the formation of PBFs (see text and Supplementary Figure 2 for more information).

We next examined the effects of culture volume and cell density on SBF formation in the microplate-based assay under static conditions as above (Figure 1). *M. xanthus* cells from an overnight culture were harvested and resuspended in fresh CYE at OD_600_ from 0.1 to 0.8. Aliquots of 75, 100, or 125 μl of these cell suspensions were placed into the microwells for SBF development for 24 h at 32°C under static conditions, followed with analysis by CV retention. The results as shown in Figure 2A indicated a general trend of increasing SBF amounts with increasing cell density up to a certain point or threshold, beyond which this trend is lost. For the 75 μl samples, the OD_600_ threshold was 0.5 as the amounts of biofilm dropped precipitously at OD_600_ of 0.6 or higher (Figure 2A). For the samples with 100 μl and 125 μl culture, the reduction in biofilms occurred at OD_600_ of 0.5 or higher (Figure 2A). Upon further examination, the decrease in SBFs at high cell density was found to coincide with the appearance of biofilms at the liquid-air interface under these experimental conditions (Supplementary Figure 2). These PBFs were removed with the culture media during the washing step before analysis by CV retention. Significant numbers of cells developed into PBFs rather than SBFs under these conditions. This explains the drastic decrease in SBF amount at higher cell densities (Figure 2A).

The formation of PBFs at the liquid-air interface has been observed and investigated for many bacteria including *Escherichia coli* and *Bacillus subtilis* (Yamamoto et al., 2011; Holscher et al., 2015; Kovacs and Dragos, 2019; Arnaouteli et al., 2021; Golub and Overton, 2021). PBFs are commonly observed on the surface of a liquid culture under static conditions in response to oxygen depletion in the liquid media (Armitano et al., 2014; Holscher et al., 2015; Kovacs and Dragos, 2019). Evidence suggests that cells may float to form aggregates at the liquid-air interface where the concentration of oxygen is the highest under these conditions (Yamamoto et al., 2011; Armitano et al., 2013, 2014; Holscher et al., 2015). These cells and their aggregates may further develop into mature PBFs by increasing the production of EPS and other biofilm matrix materials (Armitano et al., 2014; Holscher et al., 2015). As an obligate aerobe, it is perhaps not surprising that *M. xanthus* forms PBFs under static condition at high cell density. The consumption of dissolved oxygen in the media is expected to result in oxygen limitation and thus the formation of PBFs at the liquid-air interface where oxygen is more readily available. The formation of visible PBFs can therefore explain the observed reduction in *M. xanthus* SBFs at high cell densities (Figure 2A).

Indeed, the trend of SBF quantity with varying volumes of culture appeared consistent with an oxygen effect when normalized to the submerged surface areas for a sample. SBF amount (*A*_*cv*_) per cm^2^ of submerged surface area showed a seemingly decreasing trend with increasing culture volume (Figure 2B). At the same starting cell density, the higher the culture volume, the lower the *A*_*cv*_/cm^2^ value. For example, at the starting OD_600_ of 0.1, the *A*_*cv*_/cm^2^ values are 0.33 ± 0.01, 0.31 ± 0.00, and 0.27 ± 0.02 for wells with the 75, 100, and 125 μl of samples, respectively. At OD_600_ of 0.4, the values for these wells are 0.64 ± 0.06, 0.60 ± 0.02, and 0.52 ± 0.02, respectively. It can be assumed that when the depth of liquid in a microwell increases with increasing culture volumes, cells at or near the bottom of the well experience more severe oxygen limitations under static conditions. This explains the formation of PBFs at high cell density (Figures 2A,B and Supplementary Figure 2) and suggests that oxygen availability greatly influences the formation of SBFs by *M. xanthus*.

### Rotary Shaking Significantly Increases *Myxococcus xanthus* Submerged-Type Biofilms

**FIGURE 3:**
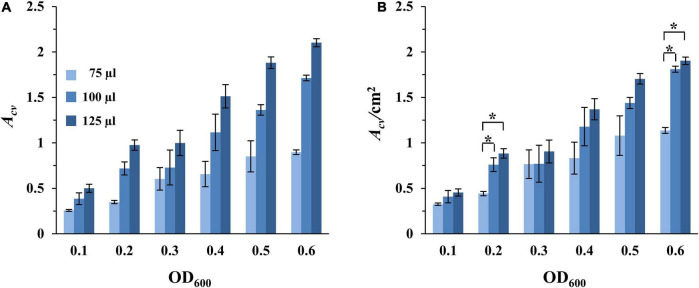
SBF formation with aeration. Experiments were conducted with DK1622 (WT) similarly as in Figure 2, except that the microplates were incubated with rotatory shaking instead of under static conditions. The amounts of SBFs were represented either by *A*_*cv*_ **(A)** or by *A*_*cv*_/cm^2^ **(B)**. Asterisks represent the initial OD_600_ values that result in statistically significant differences in normalized SBF formation between the 75 μl samples and both the 100 and 125 μl samples. Significance was determined by Student’s *t*-test, *P < 0.005.

The impact of oxygen availability on the formation of SBFs by *M. xanthus* was investigated next. Here we conducted experiments wherein cultures in the microtiter plates were aerated on a rotary shaker. For these experiments, samples were prepared as in Figure 2, except that the microtiter plates were incubated with rotary shaking. Under these conditions, the amount of SBFs per well increased steadily with increasing cell density (Figure 3A and Supplementary Figure 3). This is in stark contrast to those under static conditions where the amount of SBFs showed drastic decreases when the starting OD_600_ was equal to or greater than 0.6 (Figure 2A). Under this aerating condition, the amount of SBF continued a positive trend with increasing cell density up to OD_600_ of 0.8, the highest in our experiments (Figure 3A and Supplementary Figure 3). In addition, no PBF was observed for any of the samples, suggesting that *M. xanthus* PBF formation is sensitive to oxygen levels in the culture (Figures 2, 3).

Recall that the amounts of SBFs normalized to submerged surface areas decreased with increasing culture volume under static conditions (Figure 2B). This trend no longer holds when cultures are aerated on a rotary shaker. For wells with the same starting cell density, the amounts of SBF/cm^2^ showed no decrease as culture volume increased (Figure 3B). For example, at the starting OD_600_ of 0.1, the 75, 100, and 125 μl samples had *A*_*cv*_/cm^2^ values of 0.33, 0.41, and 0.45, respectively. At OD_600_ of 0.3, these samples gave values of 0.77, 0.77, and 0.91, respectively. In some cases, there are statistically significant increases at higher volumes (100 and 125 μl) over the 75 μl cultures. At OD_600_ of 0.2 and 0.6, for instance, the 100 and 125 μl samples produced significantly more SBFs than the 75 μl cultures (Figure 3B). The amounts of SBFs for the two higher volumes are generally not statistically different after normalization to submerged area. Although the relationship between SBF formation and culture volume under aerating conditions has yet to be fully investigated, it is clear that the inverse relationship seen under static conditions (Figure 2B) disappears when cultures are aerated through rotary shaking (Figure 3B).

Most importantly, there are significant increases in *M. xanthus* SBF when cultures are aerated in comparison with the static condition (Table 2). It is not surprising that when their counterparts formed PBFs under static conditions (shaded wells in Table 2), the corresponding samples formed significantly greater amounts of SBFs under shaking conditions. The remaining samples may be divided into two categories by culture volume. The first category includes those with 75 μl of cultures; for these samples, there does not appear to be significant differences between shaking vs. static conditions. For those with higher culture volumes (100 and 125 μl), there are generally significant increases in SBF/cm^2^ under shaking conditions (Table 2). For the 100 μl cultures, when the starting OD_600_ increased from 0.1 up to 0.4, the increases under shaking conditions ranged from 85 to 97%. For the 125 μl cultures, the increases ranged from 67% to over 160%. These results indicate that aeration through rotary shaking significantly enhanced *M. xanthus* SBF formation and it was adopted for *M. xanthus* SBF development for the remainder of the study.

**TABLE 2:** Comparison of SBFs under static and shaking conditions.

	OD_600_	0.1	0.2	0.3	0.4	0.5	0.6
75 μ l	Static	0.33 ± 0.01	0.45 ± 0.02	0.48 ± 0.03	0.64 ± 0.06	0.70 ± 0.03	0.37 ± 0.08
	Shaking	0.33 ± 0.01	0.44 ± 0.02	0.77 ± 0.16^α^	0.83 ± 0.18	1.08 ± 0.22^α^	1.14 ± 0.03
100 μ l	Static	0.31 ± 0.00	0.41 ± 0.01	0.42 ± 0.00	0.60 ± 0.02	0.28 ± 0.03	0.30 ± 0.03
	Shaking	0.41 ± 0.07	0.76 ± 0.08^β^	0.77 ± 0.20^α^	1.18 ± 0.21^α^	1.44 ± 0.06	1.81 ± 0.03
125 μ l	Static	0.27 ± 0.02	0.38 ± 0.04	0.40 ± 0.01	0.52 ± 0.02	0.24 ± 0.02	0.22 ± 0.01
	Shaking	0.45 ± 0.04^α^	0.88 ± 0.05^β^	0.90 ± 0.13^α^	1.37 ± 0.12^γ^	1.70 ± 0.06	1.90 ± 0.04

*The datasets shown here are from Figures 2A, 3A. The unit for SBF shown in the table is A_cv_/cm^2^. The first row indicates the OD_600_ of the starting culture. Shaded cells indicate PBF formation under static condition. Statistical significance between static and shaking samples are denoted with markings in the shaking cell. Statistical comparisons were made by Student’s t-test. ^α^P < 0.05, ^β^P < 0.005, ^γ^P < 0.0005.*

### Optimizing Conditions for Analyzing Vegetative Submerged-Type Biofilms of *Myxococcus xanthus*

**FIGURE 4:**
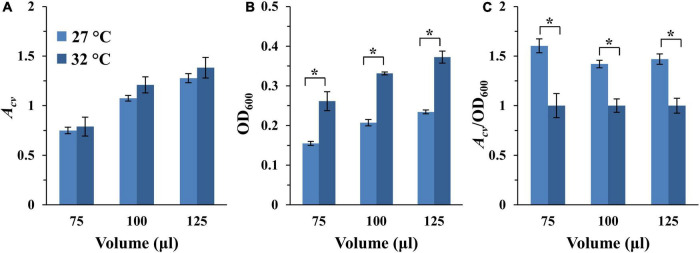
SBF formation at 27 and 32°C. The full dataset and its analysis are shown in Supplementary Figure 4. Here shows the analysis of the 75, 100, and 125 μl samples with a starting OD_600_ of 0.4 only. **(A)** SBF (*A*_*cv*_) at 27 or 32°C. **(B)** Final optical density (OD_600_) of the culture after SBF development. **(C)** Ratio of SBF amount (*A*_*cv*_) to final OD_600_ with the values for 32°C normalized to 1. The asterisk (*) indicate significant differences between the two temperatures (*P*-value < 0.05).

*Myxococcus xanthus* grows optimally at 32°C in aerated liquid culture (Janssen et al., 1977). Yet, we have observed that this bacterium produced higher levels of EPS on agar surfaces at 27°C or at room temperature (Black et al., 2017; Dye et al., 2021; unpublished data). Since EPS is a major component of the bacterial biofilm matrix, we compared the amount of SBF developed at 27 and 32°C. Here, two identical sets of experiments were initiated as in Figure 3, except one was incubated at 27°C while the other at 32°C. As shown in Figure 4A and Supplementary Figure 4A, differences in SBFs per microwell at these two temperatures are generally not statistically significant. However, because *M. xanthus* grows slower at 27°C than 32°C, the samples at 27°C were anticipated to have less growth and fewer cells. It therefore remained a possibility that a higher percentage of cells might be in SBFs at 27°C than 32°C relative to the planktonic population. We measured the optical density of the culture after biofilm development as described in section “Materials and Methods.” As expected, the OD_600_ of the culture was significantly higher at 32°C than at 27°C (Figure 4B and Supplementary Figure 4B). When SBF was normalized to the OD_600_ of the culture, it is clear that the proportion of cells in SBFs is significantly higher at 27°C than at 32°C (Figure 4C and Supplementary Figure 4C). For example, for the wells with starting OD_600_ of 0.4, the biofilm amounts by this measure are between 40 to 60% more at 27°C than at 32°C for all samples at the three volumes examined (Figure 4C). These observations indicate that *M. xanthus* cells form SBFs more readily at 27°C. Based on these analyses and previous observations, 27°C was selected as the temperature for the development of *M. xanthus* vegetative SBFs in our assay moving forward.

To finalize the remaining parameters for our microplate-based assay, we chose to use 100 μl of culture per microwell with the starting OD_600_ of 0.4. The 100 μl volume was chosen for three reasons. First, this is the most common volume in similar assays for other bacteria (Merritt et al., 2005; Kwasny and Opperman, 2010). Second, this is the volume used by Dahl et al for the analysis of *M. xanthus* SBFs previously (Dahl et al., 2011). Lastly, the difference in culture volumes per well generally did not translate into significant differences in SBF amounts under aerating condition when normalized to submerged surface area (Table 2). For the starting cell density, we took into consideration the linear range of the instrumentation (Supplementary Figure 1), aiming for an *A*_*cv*_ of ∼1.0 for DK1622 (WT) (Supplementary Figure 4). We anticipate that mutations may either enhance or diminish biofilm formation. An *A*_*cv*_ reading of ∼1.0 for the wild-type would leave room for analysis of mutants with either an increase or a decrease in biofilms formation. With 100 μl sample volume at 27°C, the starting OD_600_ of 0.4 yielded the nearest *A*_*cv*_ reading to 1.0 (Figure 4A and Supplementary Figure 4A). The following is a summary of the experimental parameters for our finalized assay for vegetative SBF of *M. xanthus*. 100 μl of a cell suspension in CYE at OD_600_ of 0.4 is inoculated into a microwell of a 96-well microplate in quadruplicates. The plate is incubated at 27°C for 24 h with rotary shaking for SBF development. The amounts of SBFs are then analyzed by CV retention using a plate reader (Supplementary Figure 1) as in similar assays for other bacteria (Christensen et al., 1985; Merritt et al., 2005; Xi and Wu, 2010).

### Exopolysaccharide, Not Type IV Pilus, Correlates With *Myxococcus xanthus* Vegetative Submerged-Type Biofilm Formation

It is known that bacterial T4P and EPS play critical roles in SBF development as adhesins and biofilm matrix materials. In *M. xanthus*, the levels of T4P and EPS are known to be intertwined in a mutual relationship. On one hand, piliation levels have been demonstrated to positively modulate EPS levels. *pilA* and *pilB* mutants, which are un-piliated, produces very low levels of EPS in both liquid culture and on agar plates (Black et al., 2006, 2009, [Bibr B8][Bibr B80]). *pilT* mutants, which are hyperpiliated because they assemble non-retractable pili (Wu et al., 1997), produces higher amounts of EPS than the wild-type in liquid culture (Black et al., 2006). On the other hand, studies suggest that EPS levels in turn can influence piliation levels. Experimental evidence supports a model wherein the retraction of T4P is triggered by interactions with EPS in *M. xanthus* (Li et al., 2003; Nudleman and Kaiser, 2004; Zhou and Nan, 2017). In other words, *M. xanthus* EPS is the preferred anchor and trigger for T4P retractions. This explains the hyperpiliated phenotypes of certain EPS^–^ mutants (Bellenger et al., 2002; Li et al., 2003; Black and Yang, 2004) because the pilus does not retract without EPS as an anchor and trigger (Li et al., 2003). In addition, it is known that EPS levels in *M. xanthus* are regulated in part by the Dif chemotaxis-like pathway (Black and Yang, 2004; Black et al., 2006, 2017; Yang et al., 2014). DifE, which resembles the CheA kinase in bacterial chemotaxis pathways, is a positive regulator of EPS. The deletion of *difE* leads to the lack of detectable EPS, absence of S motility and increased piliation levels (Bellenger et al., 2002; Li et al., 2003; Black et al., 2006). There are also negative regulators of EPS in the Dif pathway, namely, DifD and DifG, which are homologs to the chemotaxis proteins CheY and CheC, respectively (Black and Yang, 2004; Black et al., 2006). Deletions of *difD* or *difG* lead to EPS overproduction and their mutations have additive effects such that a *difD difG* double mutant produces more EPS than their respective single mutants (Black and Yang, 2004; Black et al., 2006). It is known that the Dif pathway functions downstream of T4P in EPS regulation, in part because a Δ*difD* Δ*difG* double mutation suppressed the EPS defect resulting from a *pilA* deletion (Black et al., 2006, 2017).

**FIGURE 5:**
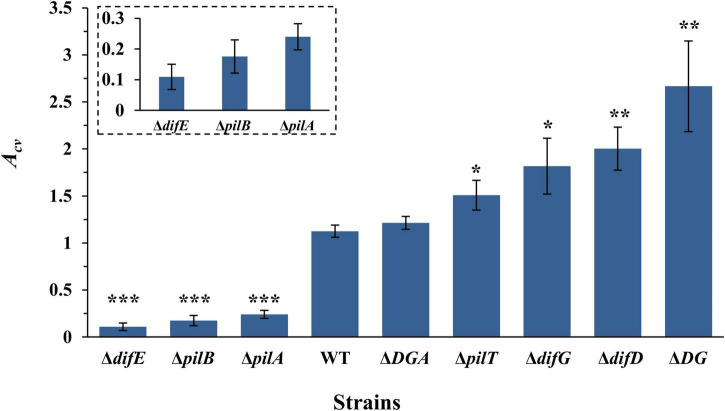
SBF formation by *M. xanthus* T4P and EPS mutants. 100 μl of a cell suspension with OD_600_ at 0.4 was placed into a microwell. SBF was develop at 27°C with rotary shaking. Shown are the average *A*_*cv*_ values with standard deviations from three independent experiments. Strains used were YZ603 (Δ*difE*), DK10416 (Δ*pilB*), YZ690 (Δ*pilA*), DK1622 (WT), YZ645 (Δ*difD* Δ*difG* Δ*pilA* or Δ*DGA)*, DK10409 (Δ*pilT*), YZ604 (Δ*difG*), YZ613 (Δ*difD*), and YZ641 (Δ*difD* Δ*difG* or Δ*DG*). Statistical difference from the WT is indicated by *P < 0.05, **P < 0.01, or ***P < 0.0001. Shown in the insert are the data for YZ603, DK10416, and YZ690 at an enlarged scale.

We analyzed the amounts of SBFs of a few *M. xanthus* mutants with altered levels of EPS and T4P using our assay. The strains here included three that were used in in earlier experiments, namely the Δ*difE* (YZ603), the Δ*pilA* (YZ690), and the WT (DK1622) strains (Figure 1). We selected six additional mutants with varying levels of EPS and T4P as established in multiple studies under different experimental conditions previously (Wu et al., 1997; Wall et al., 1998; Black et al., 2006, 2017; Wang et al., 2011; Perez-Burgos et al., 2020). These include an un-piliated Δ*pilB* mutant (DK10416) and a hyperpiliated Δ*pilT* mutant (DK10409). We also included a Δ*difG* (YZ604), a Δ*difD* (YZ613), and a Δ*difD* Δ*difG* double (YZ641) mutants. Finally, we included the Δ*difD* Δ*difG* Δ*pilA* triple mutant YZ646. This strain is un-piliated but produces similar amounts of EPS as the WT (Black et al., 2006). All of these strains were allowed to form SBFs as specified in the preceding section at 27°C, and the amounts of their SBFs were analyzed by CV retention (Figure 5).

The analysis of these results shows that *M. xanthus* SBF formation has no correlation with piliation levels under our experimental conditions. For example, both the Δ*pilT* and the Δ*difE* mutants are hyperpiliated. Yet, the SBF of the former gave a *A*_*cv*_ of 1.51, more than 10-fold higher than 0.11 for the Δ*difE* mutant (Figure 5). Similarly, the Δ*pilA*, the Δ*pilB* and the Δ*difD* Δ*difG* Δ*pilA* mutants are all un-piliated due to the deletion of either *pilA* or *pilB*. Yet the *A*_*cv*_ value for the triple mutant (1.21) is significantly higher than those for the Δ*pilA* (0.24) and the Δ*pilB* (0.18) mutants (Figure 5). Although the WT strain is piliated and the Δ*difD* Δ*difG* Δ*pilA* triple mutant is not, they produced comparable levels of SBFs in this assay. These observations clearly demonstrate that, under our experimental conditions, *M. xanthus* SBF formation has no direct correlation with piliation levels.

However, there is a clear correlation between EPS levels and SBF amounts by the different strains we examined (Figure 5). It is well established that Δ*difE*, Δ*pilA*, and Δ*pilB* mutants produce undetectable or significantly lower levels of EPS in comparison with the wild-type strains (Black et al., 2006, 2017; Yang et al., 2010). Both Δ*pilA* and Δ*pilB* mutants produced more EPS than Δ*difE* with the Δ*pilA* mutant producing slightly more ESP than a Δ*pilB* mutant (Black et al., 2006; Yang et al., 2010). It has also been demonstrated that a Δ*difD* Δ*difG* double deletion is able to suppress and restore EPS production to a Δ*pilA* mutant to about the wild-type level (Black et al., 2006). For mutants that overproduce EPS, the ascending order is Δ*pilT*, Δ*difG*,Δ*difD* and finally the Δ*difD* Δ*difG* double mutant (Black and Yang, 2004). To recap, previous studies indicate that the order of strains used here going from low to high EPS levels is YZ603 (Δ*difE*)➔DK10416 (Δ*pilB*)➔YZ690 (Δ*pilA*)➔DK1622(WT)/YZ645(Δ*difD* Δ*difG* Δ*pilA*)➔DK10409 (Δ*pilT*)➔YZ604 (Δ*difG*)➔YZ613 (Δ*difD*)➔YZ641 (Δ*difD* Δ*difG*). As shown in Figure 5, the amounts of SBFs formed by these strains followed exactly the same order as their EPS levels. These results collectively demonstrate that the level of SBF formation in *M. xanthus* under our experimental conditions tightly correlate with the amount of EPS produced by *M. xanthus* under vegetative growth. We suggest that our newly developed SBF protocol here may be utilized to conveniently and reliably quantify the relative EPS levels in *M. xanthus* under vegetative conditions in a high throughput format. Most importantly, this assay will allow further studies of *M. xanthus* SBFs to probe the mechanisms of SBF formation by an obligate aerobe adapted to living and translocating on solid surfaces in its natural environment.

## Conclusion

Here we report a microplate-based assay to analyze SBFs of *M. xanthus* under vegetative growth. This new assay has three major modifications compared with the Dahl protocol (Dahl et al., 2011). First, we demonstrated that overnight seeding in the Dahl protocol is not essential and it is therefore omitted from the new protocol for simplicity and convenience. Second, the temperature of 27°C is chosen for SBF formation because the relative cell population in SBF is significantly higher at 27°C than at 32°C; this is consistent with the observation of enhanced EPS production at 27°C or at room temperature with agar plate-based assays (Black et al., 2017; Dye et al., 2021). In retrospect, this could be the reason that 28°C was used in the Dahl protocol for overnight seeding (Dahl et al., 2011). Lastly, we introduced aeration by rotary shaking for the development of SBFs by *M. xanthus*, which is an obligate aerobe. We used our newly established protocol to examine vegetative SBF formation of various *M. xanthus* T4P and EPS mutants. The results demonstrated that the level of SBF tightly correlates with that of EPS but not of T4P, showing strains with higher EPS levels forming more SBF. Beside its use in SBF research, this assay can be utilized additionally as a convenient alternative for analyzing relative EPS levels for *M. xanthus* in a high throughput format.

## Data Availability Statement

The original contributions presented in the study are included in the article and Supplementary Material, further inquiries can be directed to the corresponding author.

## Author Contributions

KD and ZY designed the research, analyzed the data, and wrote the manuscript. KD performed the experiments. Both authors contributed to the article and approved the submitted version.

## Conflict of Interest

The authors declare that the research was conducted in the absence of any commercial or financial relationships that could be construed as a potential conflict of interest.

## Publisher’s Note

All claims expressed in this article are solely those of the authors and do not necessarily represent those of their affiliated organizations, or those of the publisher, the editors and the reviewers. Any product that may be evaluated in this article, or claim that may be made by its manufacturer, is not guaranteed or endorsed by the publisher.

## Supplementary Material

Supplementary Figure 1 Linear range of *A*_*cv*_ with CV concentrations. *A*_*cv*_ of CV solutions at indicated concentrations in parts per million (ppm) was measured by an Infinite F200 PRO plate reader. Shown are the averages with standard deviation from three independent experiments, each conducted in quadruplicates. A trendline is shown with an *R*^2^ value of 0.9977 for CV concentrations up to 60 ppm. The inset shows the dataset with CV concentrations up to 30 ppm with an *R*^2^ value of 0.9999 for the trend line.

Supplementary Figure 2 Representative images of PBF formation at the liquid-air interface. 125 μl of DK1622 (WT) cell suspension at indicated OD_600_ was placed in the microwells of a 96-well microplate in triplicates per column. The plate was incubated under static conditions at 32°C. All microwells in the top row were slightly disturbed by pipette tips to be more wrinkly to aid the visualization of PBFs.

Supplementary Figure 3 SBF formation with aeration. Shown here is the full dataset for Figure 3A.

Supplementary Figure 4 SBF formation at 27 and 32°C. The full dataset for Figure 4. Culture volumes are indicated on the top for all panels with the starting OD_600_ shown on the *X*-axis for all graphs. **(A)** SBF (*A*_*cv*_) at 27 or 32°C. **(B)** Final optical density (OD_600_) of the culture after SBF development. **(C)** Ratio of SBF amount (*A*_*cv*_) to final OD_600_ with the values for 32°C normalized to 1. The asterisk (*) indicate significant differences between the two temperatures (*P*-value < 0.05).

## References

[B1] Armitano J. Mejean V. Jourlin-Castelli C. (2013). Aerotaxis governs floating biofilm formation in *Shewanella oneidensis*. *Environ. Microbiol.* 15 3108–3118. 10.1111/1462-2920.12158 23751053

[B2] Armitano J. Mejean V. Jourlin-Castelli C. (2014). Gram-negative bacteria can also form pellicles. *Environ. Microbiol. Rep.* 6 534–544. 10.1111/1758-2229.12171 25756106

[B3] Arnaouteli S. Bamford N. C. Stanley-Wall N. R. Kovacs A. T. (2021). *Bacillus subtilis* biofilm formation and social interactions. *Nat. Rev. Microbiol.* 19 600–614. 10.1038/s41579-021-00540-9 33824496

[B4] Bahar O. Goffer T. Burdman S. (2009). Type IV Pili are required for virulence, twitching motility, and biofilm formation of *Acidovorax avenae* subsp. Citrulli. *Mol. Plant Microbe Interact.* 22 909–920. 10.1094/MPMI-22-8-0909 19589067

[B5] Bellenger K. Ma X. Shi W. Yang Z. (2002). A CheW homologue is required for *Myxococcus xanthus* fruiting body development, social gliding motility, and fibril biogenesis. *J. Bacteriol.* 184 5654–5660. 10.1128/JB.184.20.5654-5660.2002 12270823 PMC139594

[B6] Berleman J. E. Kirby J. R. (2009). Deciphering the hunting strategy of a bacterial wolfpack. *FEMS Microbiol. Rev.* 33 942–957. 10.1111/j.1574-6976.2009.00185.x 19519767 PMC2774760

[B7] Black W. P. Yang Z. (2004). *Myxococcus xanthus* chemotaxis homologs DifD and DifG negatively regulate fibril polysaccharide production. *J. Bacteriol.* 186 1001–1008. 10.1128/JB.186.4.1001-1008.2004 14761994 PMC344214

[B8] Black W. P. Wang L. Jing X. Saldana R. C. Li F. Scharf B. E. (2017). The type IV pilus assembly ATPase PilB functions as a signaling protein to regulate exopolysaccharide production in *Myxococcus xanthus*. *Sci. Rep.* 7:7263. 10.1038/s41598-017-07594-x 28779124 PMC5544727

[B9] Black W. P. Xu Q. Yang Z. (2006). Type IV pili function upstream of the Dif chemotaxis pathway in *Myxococcus xanthus* EPS regulation. *Mol. Microbiol.* 61 447–456. 10.1111/j.1365-2958.2006.05230.x 16856943

[B10] Black W. P. Xu Q. Cadieux C. L. Suh S. J. Shi W. Yang Z. (2009). Isolation and characterization of a suppressor mutation that restores *Myxococcus xanthus* exopolysaccharide production. *Microbiology (Reading)* 155(Pt 11) 3599–3610. 10.1099/mic.0.031070-0 19684067 PMC2879065

[B11] Bordeleau E. Mazinani S. A. Nguyen D. Betancourt F. Yan H. (2018). Abrasive treatment of microtiter plates improves the reproducibility of bacterial biofilm assays. *RSC Adv.* 8 32434–32439. 10.1039/c8ra06352d PMC9086168 35547717

[B12] Bretl D. J. Kirby J. R. (2016). Molecular mechanisms of signaling in *Myxococcus xanthus* development. *J. Mol. Biol.* 428 3805–3830. 10.1016/j.jmb.2016.07.008 27430596

[B13] Campos J. M. Geisselsoder J. Zusman D. R. (1978). Isolation of bacteriophage MX4, a generalized transducing phage for *Myxococcus xanthus*. *J. Mol. Biol.* 119 167–178. 10.1016/0022-2836(78)90431-x 416222

[B14] Cao P. Dey A. Vassallo C. N. Wall D. (2015). How myxobacteria cooperate. *J. Mol. Biol.* 427 3709–3721. 10.1016/j.jmb.2015.07.022 26254571 PMC4658263

[B15] Christensen G. D. Simpson W. A. Younger J. J. Baddour L. M. Barrett F. F. Melton D. M. (1985). Adherence of coagulase-negative staphylococci to plastic tissue culture plates: a quantitative model for the adherence of staphylococci to medical devices. *J. Clin. Microbiol.* 22 996–1006. 10.1128/jcm.22.6.996-1006.1985 3905855 PMC271866

[B16] Coffey B. M. Anderson G. G. (2014). Biofilm formation in the 96-well microtiter plate. *Methods Mol. Biol.* 1149 631–641. 10.1007/978-1-4939-0473-0_48 24818938

[B17] Colvin K. M. Gordon V. D. Murakami K. Borlee B. R. Wozniak D. J. Wong G. C. (2011). The pel polysaccharide can serve a structural and protective role in the biofilm matrix of *Pseudomonas aeruginosa*. *PLoS Pathog.* 7:e1001264. 10.1371/journal.ppat.1001264 21298031 PMC3029257

[B18] Costerton J. W. Lewandowski Z. Caldwell D. E. Korber D. R. Lappin-Scott H. M. (1995). Microbial biofilms. *Annu. Rev. Microbiol.* 49 711–745. 10.1146/annurev.mi.49.100195.003431 8561477

[B19] Dahl J. L. Ulrich C. H. Kroft T. L. (2011). Role of phase variation in the resistance of *Myxococcus xanthus* fruiting bodies to *Caenorhabditis elegans* predation. *J. Bacteriol.* 193 5081–5089. 10.1128/JB.05383-11 21821771 PMC3187442

[B20] Dye K. J. Vogelaar N. J. Sobrado P. Yang Z. (2021). High-throughputscreen for inhibitors of the type IV pilus assembly ATPase PilB. *mSphere* 6:e00129–21 10.1128/mSphere.00129-21 33658276 PMC8546689

[B21] Ellison C. K. Kan J. Dillard R. S. Kysela D. T. Ducret A. Berne C. (2017). Obstruction of pilus retraction stimulates bacterial surface sensing. *Science* 358 535–538. 10.1126/science.aan5706 29074778 PMC5805138

[B22] Faure L. M. Fiche J. B. Espinosa L. Ducret A. Anantharaman V. Luciano J. (2016). The mechanism of force transmission at bacterial focal adhesion complexes. *Nature* 539 530–535. 10.1038/nature20121 27749817 PMC5465867

[B23] Fiebig A. (2019). Role of *Caulobacter* cell surface structures in colonization of the air-liquid interface. *J. Bacteriol.* 201:e00064-19. 10.1128/JB.00064-19 31010900 PMC6707911

[B24] Flemming H. C. Wuertz S. (2019). Bacteria and archaea on earth and their abundance in biofilms. *Nat. Rev. Microbiol.* 17 247–260. 10.1038/s41579-019-0158-9 30760902

[B25] Flemming H. C. Wingender J. Szewzyk U. Steinberg P. Rice S. A. Kjelleberg S. (2016). Biofilms: an emergent form of bacterial life. *Nat. Rev. Microbiol.* 14 563–575. 10.1038/nrmicro.2016.94 27510863

[B26] Fu G. Bandaria J. N. Le Gall A. V. Fan X. Yildiz A. Mignot T. (2018). MotAB-like machinery drives the movement of MreB filaments during bacterial gliding motility. *Proc. Natl. Acad. Sci. U.S.A.* 115 2484–2489. 10.1073/pnas.1716441115 29463706 PMC5877941

[B27] Genevaux P. Muller S. Bauda P. (1996). A rapid screening procedure to identify mini-Tn10 insertion mutants of *Escherichia coli* K-12 with altered adhesion properties. *FEMS Microbiol. Lett.* 142 27–30. 10.1111/j.1574-6968.1996.tb08402.x 8759786

[B28] Golub S. R. Overton T. W. (2021). Pellicle formation by *Escherichia coli* K-12: role of adhesins and motility. *J. Biosci. Bioeng.* 131 381–389. 10.1016/j.jbiosc.2020.12.002 33495047

[B29] Guilhen C. Forestier C. Balestrino D. (2017). Biofilm dispersal: multiple elaborate strategies for dissemination of bacteria with unique properties. *Mol. Microbiol.* 105 188–210. 10.1111/mmi.13698 28422332

[B30] Hodgkin J. Kaiser D. (1979). Genetics of gliding motility in *Myxococcus xanthus* (Myxobacterales): two gene systems control movement. *Molec. Gen. Genet.* 171 177–191. 10.1007/bf00270004

[B31] Holscher T. Bartels B. Lin Y. C. Gallegos-Monterrosa R. Price-Whelan A. Kolter R. (2015). Motility, chemotaxis and aerotaxis contribute to competitiveness during bacterial pellicle biofilm development. *J. Mol. Biol.* 427 3695–3708. 10.1016/j.jmb.2015.06.014 26122431 PMC4804472

[B32] Islam S. T. Mignot T. (2015). The mysterious nature of bacterial surface (gliding) motility: a focal adhesion-based mechanism in *Myxococcus xanthus*. *Semin. Cell. Dev. Biol.* 46 143–154. 10.1016/j.semcdb.2015.10.033 26520023

[B33] Jakobczak B. Keilberg D. Wuichet K. Sogaard-Andersen L. (2015). Contact- and protein transfer-dependent stimulation of assembly of the gliding motility machinery in *Myxococcus xanthus*. *PLoS Genet.* 11:e1005341. 10.1371/journal.pgen.1005341 26132848 PMC4488436

[B34] Janssen G. R. Wireman J. W. Dworkin M. (1977). Effect of temperature on the growth of *Myxococcus xanthus*. *J. Bacteriol.* 130 561–562. 10.1128/jb.130.1.561-562.1977 404288 PMC235245

[B35] Kaiser D. (1979). Social gliding is correlated with the presence of pili in *Myxococcus xanthus*. *Proc. Natl. Acad. Sci. U.S.A.* 76 5952–5956. 10.1073/pnas.76.11.5952 42906 PMC411771

[B36] Keane R. Berleman J. (2016). The predatory life cycle of *Myxococcus xanthus*. *Microbiology (Reading)* 162 1–11. 10.1099/mic.0.000208 26518442

[B37] Konovalova A. Petters T. Sogaard-Andersen L. (2010). Extracellular biology of *Myxococcus xanthus*. *FEMS Microbiol. Rev.* 34 89–106. 10.1111/j.1574-6976.2009.00194.x 19895646

[B38] Kovacs A. T. Dragos A. (2019). Evolved biofilm: review on the experimental evolution studies of *Bacillus subtilis* pellicles. *J. Mol. Biol.* 431 4749–4759. 10.1016/j.jmb.2019.02.005 30769118

[B39] Kroos L. (2017). Highly signal-responsive gene regulatory network governing *Myxococcus* development. *Trends Genet.* 33 3–15. 10.1016/j.tig.2016.10.006 27916428 PMC5182100

[B40] Kuner J. M. Kaiser D. (1982). Fruiting body morphogenesis in submerged cultures of *Myxococcus xanthus*. *J. Bacteriol.* 151 458–461. 10.1128/jb.151.1.458-461.1982 6806248 PMC220259

[B41] Kwasny S. M. Opperman T. J. (2010). Static biofilm cultures of Gram-positive pathogens grown in a microtiter format used for anti-biofilm drug discovery. *Curr. Protoc. Pharmacol.* Chapter 13:Unit13A.18. 10.1002/0471141755.ph13a08s50 22294365 PMC3272335

[B42] Landini P. Antoniani D. Burgess J. G. Nijland R. (2010). Molecular mechanisms of compounds affecting bacterial biofilm formation and dispersal. *Appl. Microbiol. Biotechnol.* 86 813–823. 10.1007/s00253-010-2468-8 20165945

[B43] Lei L. Stipp R. N. Chen T. Wu S. Z. Hu T. Duncan M. J. (2018). Activity of *Streptococcus mutans* VicR Is modulated by antisense RNA. *J. Dent. Res.* 97 1477–1484. 10.1177/0022034518781765 29969955 PMC6262263

[B44] Li G. Brown P. J. Tang J. X. Xu J. Quardokus E. M. Fuqua C. (2012). Surface contact stimulates the just-in-time deployment of bacterial adhesins. *Mol. Microbiol.* 83 41–51. 10.1111/j.1365-2958.2011.07909.x 22053824 PMC3245333

[B45] Li Y. Sun H. Ma X. Lu A. Lux R. Zusman D. (2003). Extracellular polysaccharides mediate pilus retraction during social motility of *Myxococcus xanthus*. *Proc. Natl. Acad. Sci. U.S.A.* 100 5443–5448. 10.1073/pnas.0836639100 12704238 PMC154364

[B46] Lu A. Cho K. Black W. P. Duan X. Y. Lux R. Yang Z. (2005). Exopolysaccharide biosynthesis genes required for social motility in *Myxococcus xanthus*. *Mol. Microbiol.* 55 206–220. 10.1111/j.1365-2958.2004.04369.x 15612929

[B47] Maunders E. Welch M. (2017). Matrix exopolysaccharides; the sticky side of biofilm formation. *FEMS Microbiol. Lett.* 364:fnx120. 10.1093/femsle/fnx120 28605431 PMC5812517

[B48] Mauriello E. M. Mignot T. Yang Z. Zusman D. R. (2010). Gliding motility revisited: how do the myxobacteria move without flagella? *Microbiol. Mol. Biol. Rev.* 74 229–249. 10.1128/MMBR.00043-09 20508248 PMC2884410

[B49] Mercier R. Mignot T. (2016). Regulations governing the multicellular lifestyle of *Myxococcus xanthus*. *Curr. Opin. Microbiol* 34 104–110. 10.1016/j.mib.2016.08.009 27648756

[B50] Merritt J. H. Kadouri D. E. O’Toole G. A. (2005). Growing and analyzing static biofilms. *Curr. Protoc. Microbiol.* Chapter 1:Unit1B.1. 10.1002/9780471729259.mc01b01s00 18770545 PMC4568995

[B51] Merz A. J. Forest K. T. (2002). Bacterial surface motility: slime trails, grappling hooks and nozzles. *Curr. Biol.* 12 R297–R303. 10.1016/s0960-9822(02)00806-0 11967173

[B52] Mikkelsen H. Duck Z. Lilley K. S. Welch M. (2007). Interrelationships between colonies, biofilms, and planktonic cells of *Pseudomonas aeruginosa*. *J. Bacteriol.* 189 2411–2416. 10.1128/JB.01687-06 17220232 PMC1899361

[B53] Munoz-Dorado J. Marcos-Torres F. J. Garcia-Bravo E. Moraleda-Munoz A. Perez J. (2016). *Myxobacteria*: moving, killing, feeding, and surviving together. *Front. Microbiol.* 7:781. 10.3389/fmicb.2016.00781 27303375 PMC4880591

[B54] Naher J. Chowdhury S. A. Mamun A. A. Mahmud N. Shumi W. Khan R. A. (2014). A comparative study on the biofilm formation of *Enterobacter agglomerans* and *Serretia rubideae* in different environmental parameter under single culture condition. *Open J. Med. Microbiol.* 04 70–76. 10.4236/ojmm.2014.41008

[B55] Nan B. Zusman D. R. (2016). Novel mechanisms power bacterial gliding motility. *Mol. Microbiol.* 101 186–193. 10.1111/mmi.13389 27028358 PMC5008027

[B56] Nan B. McBride M. J. Chen J. Zusman D. R. Oster G. (2014). Bacteria that glide with helical tracks. *Curr. Biol.* 24 R169–R173. 10.1016/j.cub.2013.12.034 24556443 PMC3964879

[B57] Nudleman E. Kaiser D. (2004). Pulling together with type IV pili. *J. Mol. Microbiol. Biotechnol.* 7 52–62. 10.1159/000077869 15170403

[B58] O’Connor K. A. Zusman D. R. (1991a). Behavior of peripheral rods and their role in the life cycle of *Myxococcus xanthus*. *J. Bacteriol.* 173 3342–3355. 10.1128/jb.173.11.3342-3355.1991 1904432 PMC207945

[B59] O’Connor K. A. Zusman D. R. (1991b). Development in *Myxococcus xanthus* involves differentiation into two cell types, peripheral rods and spores. *J. Bacteriol.* 173 3318–3333. 10.1128/jb.173.11.3318-3333.1991 1904430 PMC207943

[B60] O’Toole G. A. Kolter R. (1998). Initiation of biofilm formation in *Pseudomonas* fluorescens WCS365 proceeds via multiple, convergent signalling pathways: a genetic analysis. *Mol. Microbiol.* 28 449–461. 10.1046/j.1365-2958.1998.00797.x 9632250

[B61] O’Toole G. Kaplan H. B. Kolter R. (2000). Biofilm formation as microbial development. *Annu. Rev. Microbiol.* 54 49–79. 10.1146/annurev.micro.54.1.49 11018124

[B62] Perez-Burgos M. Garcia-Romero I. Jung J. Schander E. Valvano M. A. Sogaard-Andersen L. (2020). Characterization of the exopolysaccharide biosynthesis pathway in *Myxococcus xanthus*. *J. Bacteriol.* 202 1–36. 10.1128/JB.00335-20 32778557 PMC7484181

[B63] Popp P. F. Mascher T. (2019). Coordinated cell death in isogenic bacterial populations: sacrificing some for the benefit of many? *J. Mol. Biol.* 431 4656–4669. 10.1016/j.jmb.2019.04.024 31029705

[B64] Redder P. Linder P. (2012). DEAD-box RNA helicases in gram-positive RNA decay. *Methods Enzymol.* 511 369–383. 10.1016/B978-0-12-396546-2.00017-6 22713329

[B65] Sanchez-Vizuete P. Dergham Y. Bridier A. Deschamps J. Dervyn E. Hamze K. (2022). The coordinated population redistribution between *Bacillus subtilis* submerged biofilm and liquid-air pellicle. *Biofilm* 4:100065. 10.1016/j.bioflm.2021.100065 35024609 PMC8732777

[B66] Shimkets L. J. (1986). Role of cell cohesion in *Myxococcus xanthus* fruiting body formation. *J. Bacteriol.* 166 842–848. 10.1128/jb.166.3.842-848.1986 3011748 PMC215203

[B67] Sydney N. Swain M. T. So J. M. T. Hoiczyk E. Tucker N. P. Whitworth D. E. (2021). The genetics of prey susceptibility to myxobacterial predation: a Review, including an investigation into *Pseudomonas aeruginosa* mutations affecting predation by *Myxococcus xanthus*. *Microb. Physiol.* 31 57–66. 10.1159/000515546 33794538

[B68] Thaxter R. (1892). On the Myxobacteriaceæ, a new order of Schizomycetes. *Bot. Gazette* 17 389–406. 10.1086/326866

[B69] Thaxter R. (1897). Contributions from the cryptogamic laboratory of Harvard University. XXXIX. Further observations on the myxobacteriaceæ. *Bot. Gazette* 23 395–411. 10.1086/327531

[B70] Thiery S. Kaimer C. (2020). The predation strategy of *Myxococcus xanthus*. *Front. Microbiol.* 11:2. 10.3389/fmicb.2020.00002 32010119 PMC6971385

[B71] van Gestel J. Vlamakis H. Kolter R. (2015). Division of labor in biofilms: the ecology of cell differentiation. *Microbiol. Spectr.* 3:MB-0002-2014. 10.1128/microbiolspec.MB-0002-2014 26104716

[B72] Velicer G. J. Vos M. (2009). Sociobiology of the myxobacteria. *Annu. Rev. Microbiol.* 63 599–623. 10.1146/annurev.micro.091208.073158 19575567

[B73] Wadhwa N. Berg H. C. (2021). Bacterial motility: machinery and mechanisms. *Nat. Rev. Microbiol*. 20 161–173. 10.1038/s41579-021-00626-4 34548639

[B74] Wall D. Wu S. S. Kaiser D. (1998). Contact stimulation of Tgl and type IV pili in *Myxococcus xanthus*. *J. Bacteriol.* 180 759–761. 10.1128/JB.180.3.759-761.1998 9457887 PMC106951

[B75] Wang J. Hu W. Lux R. He X. Li Y. Shi W. (2011). Natural transformation of Myxococcus xanthus. *J Bacteriol* 193 2122–2132. 10.1128/JB.00041-11 21378184 PMC3133062

[B76] Wu S. S. Wu J. Kaiser D. (1997). The *Myxococcus xanthus pilT* locus is required for social gliding motility although pili are still produced. *Mol. Microbiol.* 23 109–121. 10.1046/j.1365-2958.1997.1791550.x 9004225

[B77] Xi C. Wu J. (2010). dATP/ATP, a multifunctional nucleotide, stimulates bacterial cell lysis, extracellular DNA release and biofilm development. *PLoS One* 5:e13355. 10.1371/journal.pone.0013355 20976227 PMC2954796

[B78] Yamamoto K. Arai H. Ishii M. Igarashi Y. (2011). Trade-off between oxygen and iron acquisition in bacterial cells at the air-liquid interface. *FEMS Microbiol. Ecol.* 77 83–94. 10.1111/j.1574-6941.2011.01087.x 21395624

[B79] Yang Z. Li C. Friedrich C. Søgaard-Andersen L. (2014). “Type IV pili and exopolysaccharide-dependent motility in *Myxococcus xanthus*,” in *Myxobacteria: Genomics, Cellular and Molecular Biology*, eds Yang Z. Higgs P. I. (London: Caister Academic Press), 183–198.

[B80] Yang Z. Lux R. Hu W. Hu C. Shi W. (2010). PilA localization affects extracellular polysaccharide production and fruiting body formation in *Myxococcus xanthus*. *Mol. Microbiol.* 76 1500–1513. 10.1111/j.1365-2958.2010.07180.x 20444090 PMC2935901

[B81] Zhang Y. Ducret A. Shaevitz J. Mignot T. (2012). From individual cell motility to collective behaviors: insights from a prokaryote, *Myxococcus xanthus*. *FEMS Microbiol. Rev.* 36 149–164. 10.1111/j.1574-6976.2011.00307.x 22091711

[B82] Zhou T. Nan B. (2017). Exopolysaccharides promote *Myxococcus xanthus* social motility by inhibiting cellular reversals. *Mol. Microbiol.* 103 729–743. 10.1111/mmi.13585 27874229

[B83] Zusman D. R. Scott A. E. Yang Z. Kirby J. R. (2007). Chemosensory pathways, motility and development in *Myxococcus xanthus*. *Nat. Rev. Microbiol.* 5 862–872. 10.1038/nrmicro1770 17922045

