# Supplementary figures and images for: Analysis of Myxococcus xanthus Vegetative Biofilms With Microtiter Plates
Source: Front Microbiol. 2022 Apr 29;13:894562. doi: 10.3389/fmicb.2022.894562 (PMC9100584; doi:10.3389/fmicb.2022.894562)

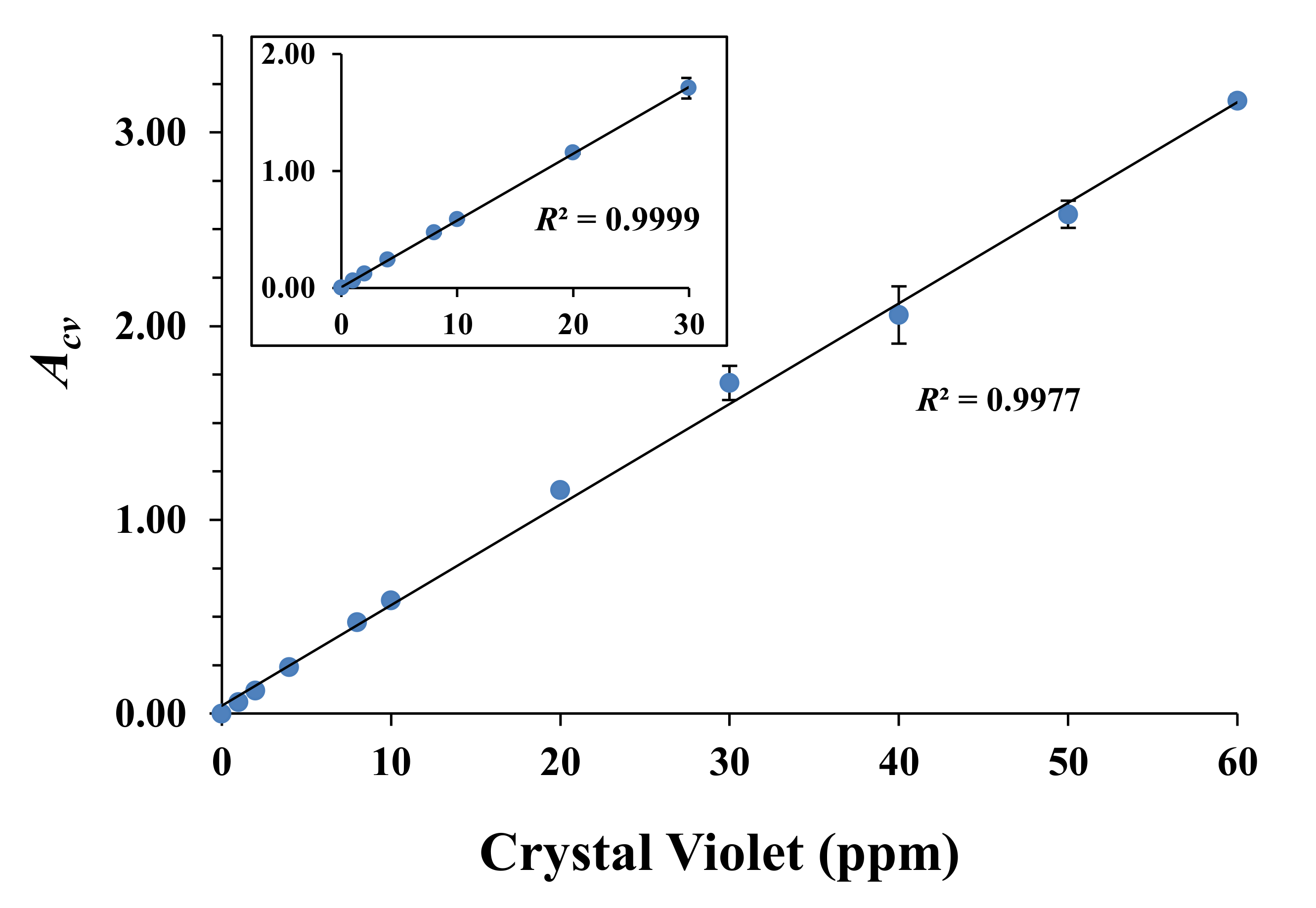

Supplement: Supplementary Figure 1 — Linear range of Acv with CV concentrations. Acv of CV solutions at indicated concentrations in parts per million (ppm) was measured by an Infinite F200 PRO plate reader. Shown are the averages with standard deviation from three independent experiments, each conducted in quadruplicates. A trendline is shown with an R2 value of 0.9977 for CV concentrations up to 60 ppm. The inset shows the dataset with CV concentrations up to 30 ppm with an R2 value of 0.9999 for the trend line. [file Image_1.TIFF]

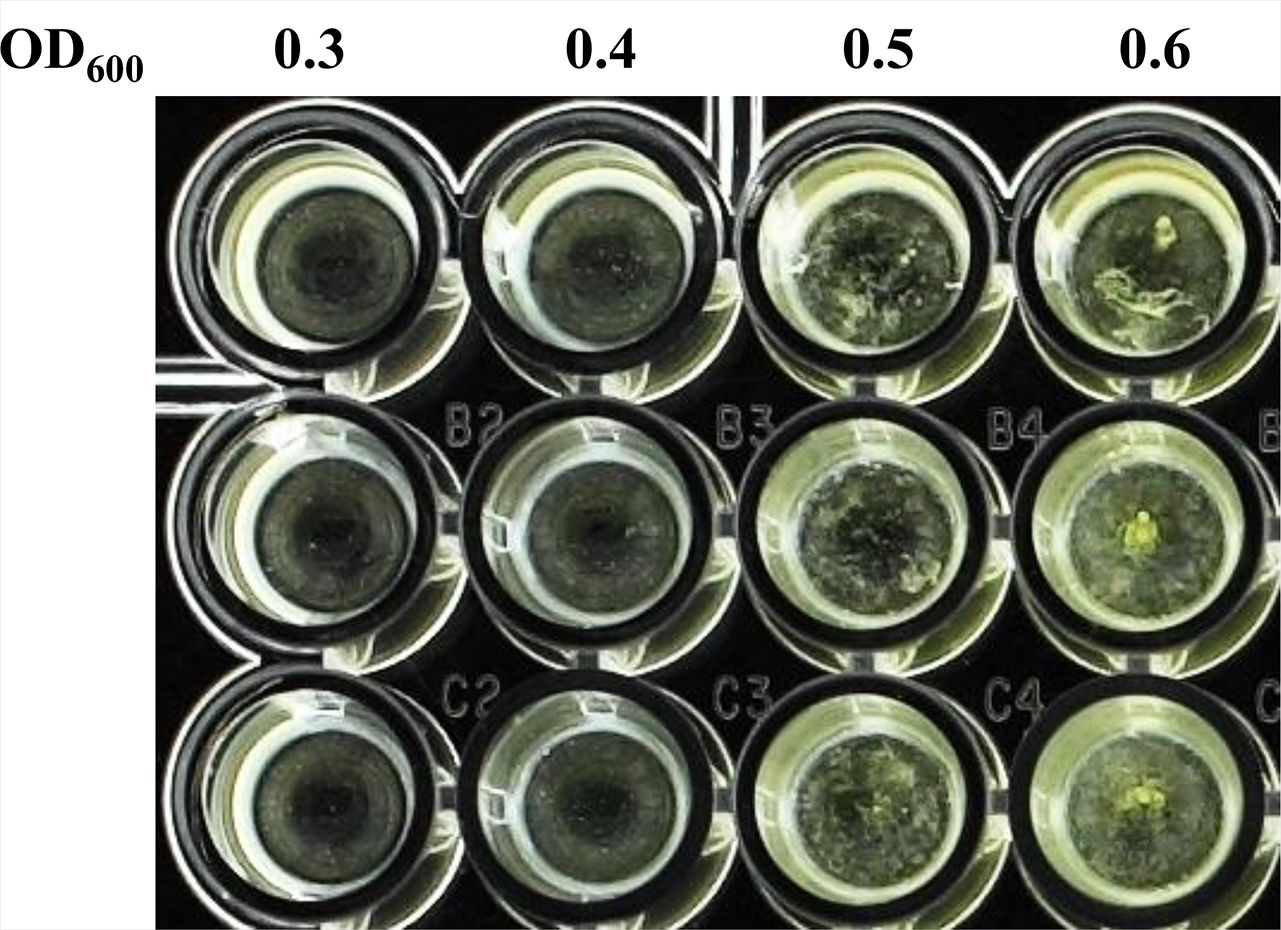

Supplement: Supplementary Figure 2 — Representative images of PBF formation at the liquid-air interface. 125 μl of DK1622 (WT) cell suspension at indicated OD600 was placed in the microwells of a 96-well microplate in triplicates per column. The plate was incubated under static conditions at 32°C. All microwells in the top row were slightly disturbed by pipette tips to be more wrinkly to aid the visualization of PBFs. [file Image_2.TIFF]

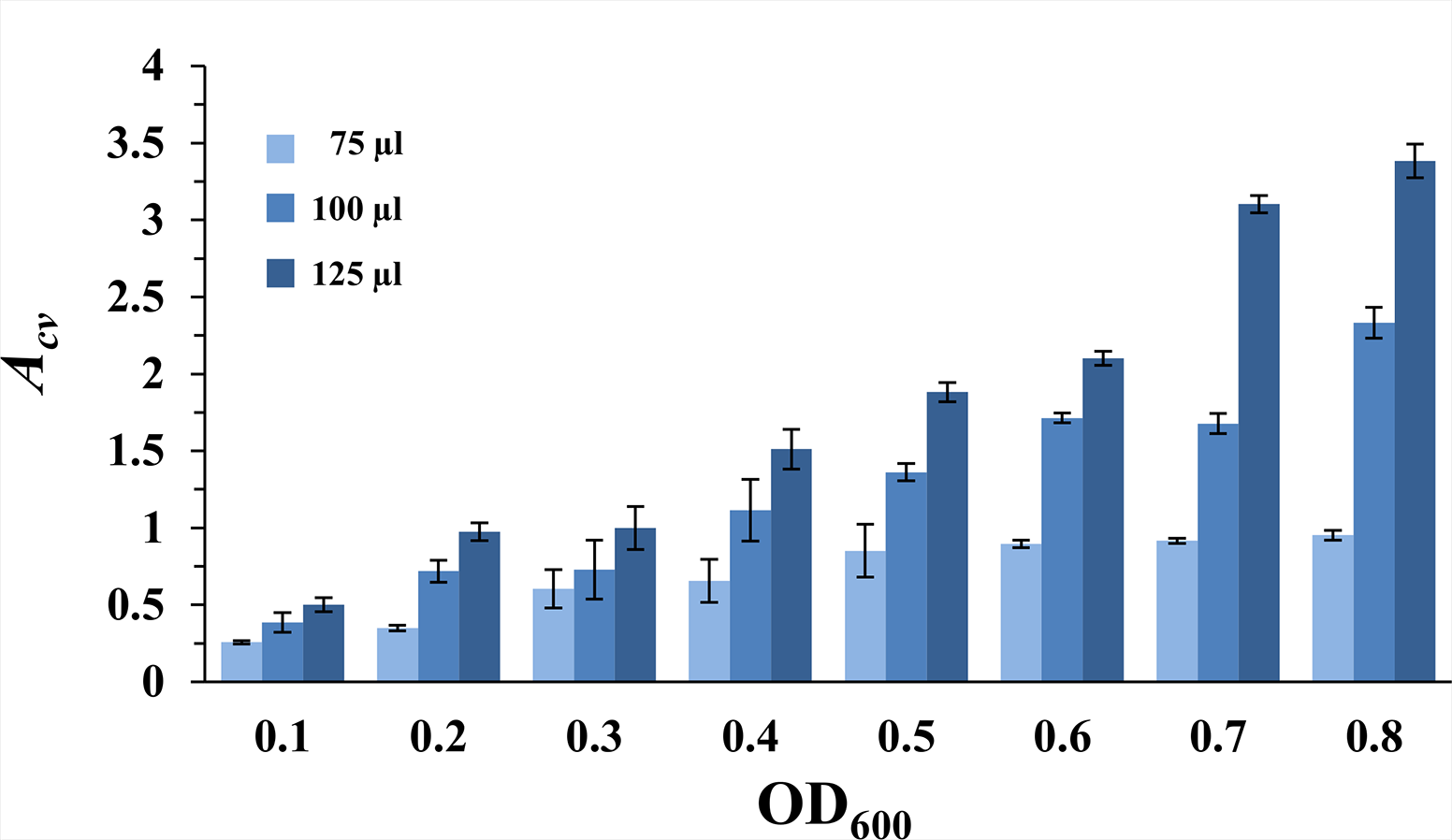

Supplement: Supplementary Figure 3 — SBF formation with aeration. Shown here is the full dataset for Figure 3A. [file Image_3.TIFF]

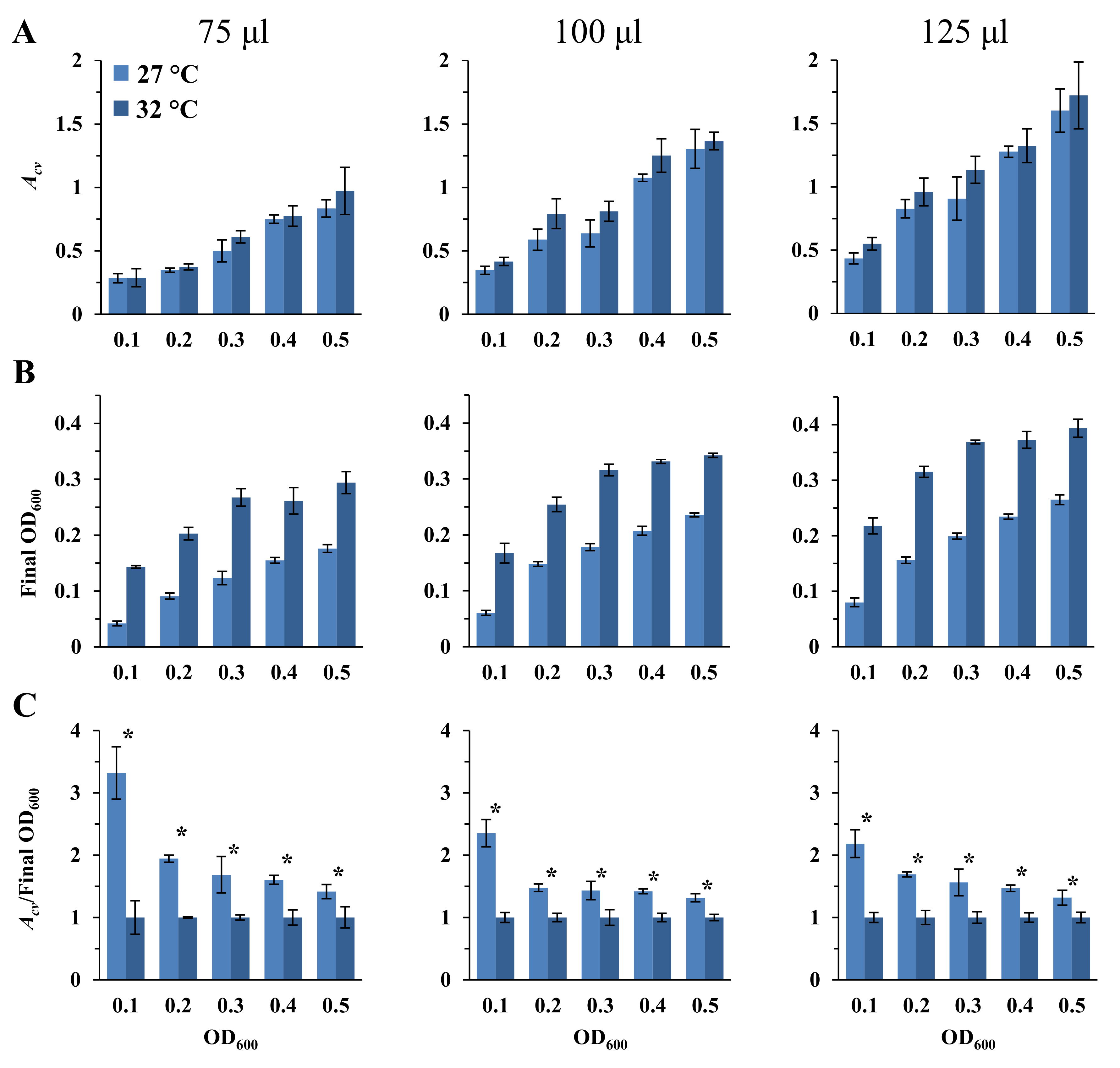

Supplement: Supplementary Figure 4 — SBF formation at 27 and 32°C. The full dataset for Figure 4. Culture volumes are indicated on the top for all panels with the starting OD600 shown on the X-axis for all graphs. (A) SBF (Acv) at 27 or 32°C. (B) Final optical density (OD600) of the culture after SBF development. (C) Ratio of SBF amount (Acv) to final OD600 with the values for 32°C normalized to 1. The asterisk (*) indicate significant differences between the two temperatures (P-value < 0.05). [file Image_4.JPEG]
